# Efficacy and safety of the C-Qur™ Film Adhesion Barrier for the prevention of surgical adhesions (CLIPEUS Trial): study protocol for a randomized controlled trial

**DOI:** 10.1186/1745-6215-15-378

**Published:** 2014-09-26

**Authors:** Martijn WJ Stommel, Chema Strik, Richard PG ten Broek, Harry van Goor

**Affiliations:** Department of Surgery, Radboud University Medical Center, P.O. Box 9101, 6500 HB Nijmegen, The Netherlands

**Keywords:** adhesions, adhesion prevention, anti-adhesive barrier, C-Qur™ film, colorectal surgery

## Abstract

**Background:**

Adhesions develop in over 90% of patients after intra-abdominal surgery. Adhesion barriers are rarely used despite the high morbidity caused by intra-abdominal adhesions. Only one of the currently available adhesion barriers has demonstrated consistent evidence for reducing adhesions in visceral surgery. This agent has limitations through poor handling characteristics because it is sticky on both sides. C-Qur™ Film is a novel thin film adhesion barrier and it is sticky on only one side, resulting in better handling characteristics. The objective of this study is to assess efficacy and safety of C-Qur™ Film to decrease the incidence of adhesions after colorectal surgery.

**Methods/Design:**

This is a prospective, investigator initiated, randomized, double-blinded, multicenter trial. Eligible patients undergoing colorectal resection requiring temporary loop ileostomy or loop/split colostomy by laparotomy or hand assisted laparoscopy will be included in the trial. Before closure, patients are randomized 1:1 to either the treatment arm (C-Qur™ Film) or control arm (no adhesion barrier). Patients will return 8 to 16 weeks post-colorectal resection for take down of their ostomy. During ostomy takedown, adhesions will be evaluated for incidence, extent, and severity. The primary outcome evaluation will be assessment of adhesions to the incision site. It is hypothesized that the use of C-Qur™ Film underneath the primary incision reduces the incidence of adhesion at the incision by 30%. To demonstrate 30% reduction in the incidence of adhesions, a sample size of 84 patients (32 + 10 per group (25% drop out)) is required (two-sided test, α = 0.05, 80% power).

**Discussion:**

Results of this study add to the evidence on the use of anti-adhesive barriers in open and laparoscopic ‘hand-assisted’ colorectal surgery. We chose incidence of adhesions to the incision site as primary outcome measure since clinical outcomes such as small bowel obstruction, secondary infertility and adhesiolysis related complications are considered multifactorial and difficult to interpret. Incidence of adhesions at repeat surgery is believed to be the most valuable surrogate endpoint for clinically relevant adhesion prevention, since small bowel obstruction and adhesiolysis at repeat surgery are not likely to occur when complete adhesion reduction in a patient is accomplished.

**Trial registration:**

ClinicalTrials.gov Identifier NCT01872650, registration date 6 June 2013.

## Background

Postoperative adhesions develop in more than 90% of patients undergoing an intra-abdominal surgical procedure [1]. These adhesions are known to cause small bowel obstruction, secondary infertility and pain [2–4]. At repeat abdominal surgery, intra-abdominal adhesions necessitate adhesiolysis, leading to increased operating times and substantial risk of intra- and postoperative complications [5, 6]. The clinical implications of adhesions carry a significant health and socioeconomic burden [7, 8].

Adhesions are fibrous bands that connect tissue surfaces where anatomical connections do not normally exist. Adhesions are formed after a tissue surface has been injured (abrasion, desiccation, dissection, *etcetera*) and the subsequent process of fibrinolysis is incomplete [9]. At present, there are several products on the market to combat postsurgical adhesion formation. These are broad coverage adhesion barriers, generally consisting of liquids, like icodextrin 4% (Adept™, Baxter, Deerfield, IL, USA) adhesion reduction solution. These materials may limit adhesion formation by minimizing tissue insult during the surgical procedure when used as an intra-operational lavage. At the conclusion of the surgical procedure, instillation of large volumes of liquid acts to separate tissue surfaces by hydroflotation, limiting tissue-tissue contact. Broad coverage adhesion prevention has the distinct advantage of allowing the surgeon to treat many areas of the intraperitoneal space at once. A disadvantage is the lack of control giving adhesion prevention at sites at risk for complications of adhesion prevention such as an anastomosis. In addition, side effects of abdominal distension and vulvar swelling are commonly encountered in the use of liquids [10]. The second group of barriers consists of local coverage adhesion barriers: films, sprays, or gels that are applied directly to adhesiogenic tissue surfaces to act as a physical barrier that blocks tissue-tissue contact. The barrier is generally only effective when it remains at the site of treatment. This requires the barrier to either be sutured or glued in place or to have tissue adherent properties. All local coverage adhesion barriers require the surgeon to anticipate where adhesions are likely to occur and to apply the barrier directly to those sites. These generally include sites where the peritoneum was interrupted during the surgical procedure, such as under a laparotomy incision to minimize adhesions between the incision site and the viscera [11].

Despite the frequent occurrence of adhesions after surgery and the availability of different adhesion barriers, these barriers are rarely used by general surgeons. This discrepancy might be explained by an underestimation of the impact of adhesions [12]. Also, of all the clinically available adhesion barriers, only HA/CMC (hyaluronate/carboxymethylcellulose) barrier film (Seprafilm™, Sanofi, Paris, France) has demonstrated consistent evidence for reducing adhesions in visceral surgery. Seprafilm™ has limitations through poor handling characteristics because it is sticky on both sides. In a large, prospective, randomized controlled multicenter study on the safety of Seprafilm™, significantly more anastomotic leak and leak-associated complications occurred in the Seprafilm™ group due to the wrapping of Seprafilm™ around an anastomosis [13]. A subgroup analysis of control patients and patients without (593 patients) Seprafilm™ wrapped around the anastomosis showed no significant difference. Therefore, wrapping the suture or staple line of a fresh bowel anastomosis should be avoided.

Atrium (Atrium Medical Corporation, Maquet Gentinge Group, Hudson, NH, USA) has developed and manufactures C-Qur™ Film Adhesion Barrier, a novel thin film adhesion barrier for intraperitoneal use in general surgeries. The C-Qur™ Film has been approved for use in humans and received a CE mark on 13 May 2011 (CE number 10123365). The evidence on efficacy and safety of the C-Qur film originates from research on the C-Qur mesh, a mesh with an omega-3 fatty acid coating used for patients with an abdominal ventral hernia. The C-Qur mesh was found to be safe in intraperitoneal use and reduces adhesions to the mesh [14]. The C-Qur™ Film falls into the category of local coverage adhesion barriers. The C-Qur™ Film is an adhesion barrier consisting of a non-adhesive omega-3 fatty acid layer on one side and a Na-CMC (sodium-carboxymethylcellulose) tissue adherent coating on the other side. It is fully resorbable and designed to adhere to the site of treatment for a time that is sufficient to minimize postsurgical adhesion formation and clearance from the site of treatment within approximately 60 days. In contrast to Seprafilm™, The C-Qur™ Film is a one-sided adherent, resulting in good handling characteristics. It offers the potential patient benefits of reduced adhesion formation and corresponding reduction of small bowel obstruction, secondary infertility, pain and adhesiolysis at repeat surgery. The aim of this study is to assess the efficacy and safety of C-Qur™ Film to decrease the incidence of adhesions after colorectal surgery.

## Methods/Design

The CLIPEUS trial is registered with the ClinicalTrials.gov Identifier NCT01872650. The protocol was ethically approved by the official Independent Review Board Nijmegen (2013/470) and registered nationally (NL45940.091.13) [15].

### Design

The CLIPEUS trial is a prospective, investigator-initiated, randomized controlled, double-blinded, multicenter trial. Treatment with the C-Qur™ film adhesion barrier will be compared with no treatment with an adhesion barrier in patients undergoing colorectal surgery with temporary diverting ostomy. The surgeons who have agreed to participate in the study and perform the index procedures will be trained in the placement of C-Qur™ Film and in adhesion mapping.

Patients will be included at the outpatient clinics of the participating centers (Radboud University Medical Center, Nijmegen; Catharina Hospital, Eindhoven; Atrium Medical Center, Heerlen; Maxima Medical Center, Veldhoven; Gelderse Vallei Hospital, Ede) by the treating surgeons. During the operation, when the definite decision to create a temporary ostomy is made, patients are randomized 1:1 to either the treatment arm (C-Qur™ film) or the control arm (standard treatment: no adhesion barrier, no placebo). In patients assigned to the treatment arm, the C-Qur™ Film must be applied beneath the incision. The C-Qur™ film can also be applied to other areas considered to be adhesiogenic (for example, the dissection site and ostomy site, but not around the anastomosis). The number of C-Qur™ Film sheets placed is limited to a maximum area of coverage of 774 cm^2^ (Table 1). Patients will return 8 to 16 weeks post-colorectal resection to have their diverting ostomy taken down. During the takedown, the incidence, extent and severity of the adhesions will be evaluated.

**Table 1:** **Maximum area of coverage and number of sheets**

Code	Size (cm)	Maximum	
		Area (cm ^2^)	Number of sheets
32024	7.5 × 10.0	774	10
32025	7.5 × 12.5	774	8
32029	10.0 × 10.0	774	7
32031	12.5 × 15.0	774	4

### Patients

Patients aged 18 years or older who require open or hand-assisted laparoscopic colonic or rectal resection for colorectal disease with the formation of a temporary diverting loop ileostomy or colostomy and a planned closure within 8 to 16 weeks and who visit the outpatient clinic at one of the participating centers will be invited to participate in this trial.

Inclusion criteria are an incision of 6 cm or longer in case of hand-assisted laparoscopy and signed informed consent.

Exclusion criteria include the following: pregnancy, patients for whom it is known prior to the initial procedure that loop ileostomy or colostomy closure between 8 and 16 weeks is not feasible, active intra-abdominal infection such as peritonitis, abdominal abscess, anastomotic leakage or fistula (interloop abscess in the resection specimen is not an exclusion criterion), endometriosis, known allergies to any component of the C-Qur Film device, an additional procedure at the time of loop ileostomy or colostomy takedown deemed interfering with adhesion assessment by the treating surgeon, intended use of intraoperative lavage/irrigation with any anti-adhesion solutions other than lactated ringers and/or saline (for example, dextran, heparin, corticosteroids, icodextrin, any other irrigant that is believed to have anti-adhesion properties) or an adhesion barrier other than C-Qur Film™, (planned) administration of systemic agents with the intention to prevent adhesion formation within 30 days prior to the index procedure, planned chemotherapy and/or abdominal radiotherapy between index surgery and loop ileostomy or colostomy takedown, use of immune system suppressants deemed by the surgeon to interfere with wound healing (patients taking daily doses of corticosteroids exceeding 20 mg within the prior 30 days are to be excluded; patients requiring perioperative corticosteroid supplementation are not to be excluded), impaired immune system function or coagulation disorders deemed by the surgeon to interfere with wound healing, a known history of severe multiple drug allergies, a life expectancy of less than 6 months because of a medical condition or disease state, a medical condition or other serious condition that will interfere with compliance and/or ability to complete this study protocol or patients who in the opinion of the investigator would not be a good candidate for enrolment, or participation in a study of another investigational device or drug.

### Intervention

**Figure 1:**
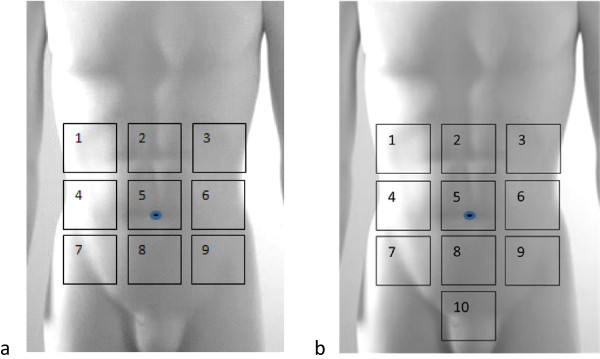
**Segments of the abdominal wall and segments of the abdominal cavity. a**. The nine segments of the abdominal wall; **b**. The ten segments of the abdominal cavity.

All patients will undergo a colorectal resection with the creation of a temporary loop ileostomy or loop or split colostomy. During this index operation, adhesions, if any, will be mapped. The incidence, location, extent and severity and any treatment of adhesions will be noted. For quantifying the extent of adhesions to the abdominal wall and between organs, the abdominal wall is divided into nine segments and the abdominal cavity into ten segments (Figure 1). Severity of the adhesions will be classified according to the Zühlke classification (Table 2) [16]. Classification of operative wounds based on degree of microbial contamination, number of serosal injuries and number of inadvertent enterotomies will also be noted in the source notes and CRF (case report form).

**Table 2:** **Adhesion classification system according to Zühlke** [16]

Score	Observation
0	No adhesions
1	Adhesions that are filmy and easy to separate by blunt dissection
2	Adhesions where blunt dissection is possible but sharp dissection necessary, beginning vascularization
3	Lysis of adhesions possible by sharp dissection only, clear vascularization
4	Lysis of adhesions possible by sharp dissection only, organs strongly attached with severe adhesions, damage of organs hardly preventable

For patients randomized to the C-Qur™ Film arm, the C-Qur™ Film must be applied to the viscera underneath the primary or specimen retrieval incision in case of hand-assisted laparoscopy. Preferably, the C-Qur™ Film is also applied to other areas considered to be adhesiogenic, such as the peritoneal dissection planes and the ileum or colon at the ostomy site. The number of C-Qur™ Film sheets placed in the abdomen is limited to a maximum area of coverage of 774 cm^2^. In case sheets of the largest size (15 cm × 12.5 cm) are used, the maximum is four sheets (Table 1). The application of C-Qur™ Film directly to a fresh bowel anastomosis is not allowed.

### Control arm

Patients who are not allocated to the treatment group will receive standard treatment, which means no C-Qur™ Film or any other treatment considered to have anti-adhesion properties (for example, corticosteroids, dextran, heparin, icodextrin, *etcetera*) and no placebo will be used.

### Outcome measures

The primary outcome measure will be the incidence of adhesions to the primary incision site or the specimen retrieval incision in the case of hand-assisted laparoscopy.

Secondary outcome measures on effectiveness are the extent and severity of adhesions to the primary incision site or specimen retrieval incision, incidence, extent and severity of adhesions at the loop ileostomy or loop/split colostomy site and of adhesions at areas potentially injured during the initial procedure, duration of ileostomy or loop/split colostomy takedown from the start of the takedown to the time the bowel is repositioned in the abdomen, percentage (%) of abdominal wall with adhesions, number of C-Qur™ barrier films used, sizes of C-Qur™ barrier films used, areas treated with C-Qur™ Films (underneath incision, ostomy bowel loops, other areas injured during initial procedure and considered adhesiogenic), reason for not placing film in one or more of the areas considered to be adhesiogenic, incidence of chronic abdominopelvic pain, incidence of other gastrointestinal complaints, quality of life as assessed by Short Form-36 and DASI, and total direct health care costs (30 days-in-hospital healthcare costs during both hospital stays).

Secondary outcome measures on safety are incidence of postoperative complications. Complications are divided into surgical and medical complications. Surgical complications are superficial incisional surgical site infections, deep incisional surgical site infections, anastomotic leakage, intra-abdominal abscess, peritonitis, unexplained fever, fascia dehiscence, wound dehiscence, postoperative hemorrhage, and postoperative ileus (POI). Medical complications are pulmonary embolism, pneumonia, urinary tract infection, sepsis, and death.

Other secondary outcome measures are reoperations, number of re-laparotomies, number of inserted central venous lines, other re-operations, first postoperative day of oral food intake, parenteral feeding, number of days parenteral feeding required, tube feeding, number of days tube feeding required, first postoperative day of passing flatus, first postoperative day of passing stool, usage of pain medication, daily VAS (Visual Analog Scale) scores, hospital stay, Intensive Care Unit stay, Recovery Unit stay, readmissions within 30 days after discharge, and in-hospital mortality.

### Randomization and blinding

Intra-operatively, the treating surgeon judges whether the patient is eligible for definite inclusion. Prior to formation of the temporary loop ileostomy, loop or split colostomy, included patients will be randomized. Patients will be randomized in a 1:1 fashion to the C-Qur™ Film arm (treatment arm) or the control arm. The randomization will be stratified by operative technique (open or hand-assisted laparoscopy). The allocation sequence will be computer-generated through the web-based program Alea©. A researcher who does not participate in the data analysis will manage the randomization process. This way, the researcher who will perform the data analysis is blinded from treatment allocation. Concealment of treatment allocation and blinding of the surgeon performing the index procedure is not possible because the patient will either get the intervention or does not get an intervention at all. Randomization will be noted in the operation reports, allocation will not.

The patient and doctor at the surgical ward who will complete the case report form during the post-operative period is blinded from treatment allocation. To achieve blinding during the data collection of outcome parameters, the surgeon who will perform the ostomy takedown procedure, will not be the same surgeon as the one who performed the index procedure. Because of the absence of clinical notes regarding allocation, however, the initial surgeon is allowed to do the ostomy takedown in case of man-power or organizational problems.

During the postoperative period after the ostomy takedown, the physician at the ward, who will collect the data regarding the post-operative parameters, will be blinded from the treatment allocation of the patient. The patient will stay blinded for the received treatment.

### Unblinding protocol

A Data Safety Monitoring Board (DSMB) meeting for safety assessment will be scheduled once every 15 patients have been enrolled. The study will be deblinded when there is a higher mortality rate or incidence of surgical complications than can be expected from data found in the literature. The deblinding will be performed by the same researcher who performs the randomization procedure.

Patients will be informed on request about the performed procedure only after completing the quality-of-life questionnaires 1 year postoperatively.

### Data recording and follow-up

All included patients will preoperatively fill in two questionnaires on quality of life (SF-36, short-form 36, and DASI, duke activity status index) and one questionnaire on gastro-intestinal complaints (GIC). Preoperatively, information on the following will be collected: age, sex, weight, height, American Society of Anesthesiologists (ASA) classification, smoking status, Revised Cardiac Risk Index (RCRI), chronic obstructive pulmonary disease (COPD)/asthma, diabetes mellitus, primary clinical diagnosis, medical/surgical history, number of previous laparotomies, type of previous laparotomies, number of previous laparoscopies, type of previous laparoscopies, and medication usage. A physical exam will be performed, which includes vital signs and laboratory collection (to include C-reactive protein (CRP), chemistry, hematology and coagulation) and a pregnancy test if the patient is premenopausal.

Classification of operative wounds based on the degree of microbial contamination, the number of serosal injuries and the number of inadvertent enterotomies will be noted in the source notes and CRF.

The type of abdominal closure (layered or mass fascia closure) and suture material used will be noted in the source notes and CRF.

Patients in both groups will receive the same postoperative treatment. Until patients are discharged, the following parameters will be collected: postoperative complications (superficial incisional surgical site infections, deep incisional surgical site infections, anastomotic leakage, intra-abdominal abscess, peritonitis, unexplained fever, fascia dehiscence, wound dehiscence, postoperative hemorrhage, postoperative ileus (POI), pulmonary embolism, pneumonia, urinary tract infection, sepsis, and death), reoperations, first post-operative day of oral food intake, parenteral feeding, number of days parenteral feeding required, tube feeding, number of days tube feeding required, first post-operative day of passing flatus, first post-operative day of passing stool, usage of medication including pain medication, daily VAS-scores, hospital stay, Intensive Care Unit stay and Recovery Unit stay.

Patients will return 8 to 16 weeks after index surgery to have their ostomy taken down. Clinical follow-up to obtain update(s) on adverse events (AE’s), adhesion-related events, changes in concomitant medications, the presence/absence of surgical site infection (SSI) and the type of SSI (if applicable) will be done during the admission for ileostomy/colostomy takedown. Weight and vital signs will be noted and the following laboratory investigations will be repeated: CRP, sodium (Na), potassium (K), urea, creatinine, hemoglobin, white blood cell count, platelet count, international normalized ratio (INR) and prothrombin time (PT).

**Figure 2:**
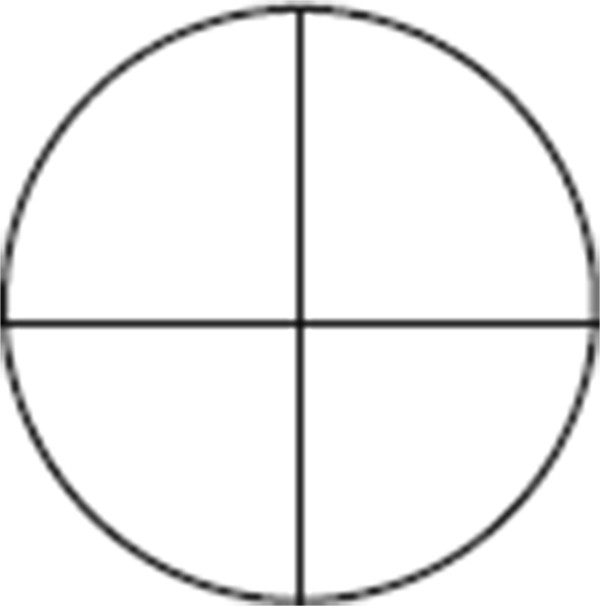
**Quadrants of the ostomy site.**

Patients in both groups will undergo the same procedure for ostomy closure. To evaluate the adhesions at the loop ileostomy/colostomy site, the incidence, severity and extent of adhesions around the ostomy have to be evaluated during takedown. The time required for takedown of the ostomy is defined as the time from start of the takedown to the time the bowel is repositioned in the abdomen, and this time will be noted on the CRF. The severity of adhesions will be scored according to the Zühlke classification (see Table 2). To assess the extent of adhesions, the ostomy is divided in four quadrants (Figure 2). The extent of adhesions is scored as the number of quadrants containing adhesions (Table 3). After the ileostomy/colostomy takedown is completed, the surgeon will introduce a laparoscope at the ostomy site and evaluate the incidence, extent and severity of adhesions at the incision site and at other areas potentially injured (and covered) during the initial procedure. The severity will be scored according to the Zühlke classification (see Table 2). The extent of adhesions underneath the incision site will be scored through estimation of the area covered by adhesions as a percentage of the total area underneath the incision. The incidence and severity of adhesions at other areas potentially injured during the initial procedure will be scored according to the Zühlke classification (see Table 2).

**Table 3:** **Classification to score the extent of adhesions covering the stoma**

Score	Observation
0	No adhesions
1	Adhesions present on up to one quadrant of the stoma circle
2	Adhesions present on two quadrants of the stoma circle
3	Adhesions present on three quadrants of the stoma circle
4	Adhesions present on all four quadrants of the stoma circle

One year after randomization, patients in the treatment and control arm will be approached to fill in the same questionnaires on quality of life (SF-36 and DASI) and gastrointestinal complaints (GIC) as preoperatively.

### Ethics and informed consent

This study is conducted in concordance with the principles of the Declaration of Helsinki [17] and Good Clinical Practice guidelines. The protocol was ethically approved by the official Independent Review Board Nijmegen (2013/470) and registered nationally (NL45940.091.13) [15]. A Data and Safety Monitoring Board (DSMB) is established to perform safety surveillance and to perform interim analysis on the safety data, as described.

Patients will be screened in the outpatient clinic for participation in this study by a surgeon in one of the hospitals that will contribute to this study. Each patient must meet the inclusion/exclusion criteria of this trial. All potentially eligible patients are required to sign an informed consent after careful consideration. Patients have to sign the informed consent prior to the index procedure.

### Analysis and sample size

It is hypothesized that the use of the C-Qur™ Film underneath the primary incision reduces the incidence of adhesion at the incision by 30%. This hypothesis is based on the supporting documentation for Seprafilm™ (treatment) versus Control (no treatment) with an adhesion rate of 94% in the control group and 64% in the treatment group [18–22]. We estimate a total drop out of 25% in this study, comprising 10% regular drop out and 15% drop out because of anastomotic leakage. A 15% drop out because of anastomotic leakages is chosen because it is the upper limit of the incidence of this complication. Assuming that the C-Qur™ Film group performs similarly to Seprafilm™, a total of 84 patients, (32 + 10 per group (25% drop out)) would be needed in a randomized study, with 80% power and two-sided alpha = 0.05 in order to detect a 30% reduction in incidence of adhesions.

### Statistics

In general, for continuous variables, the mean, standard deviation, median, IQR, minimum and maximum values will be presented. Groups will be compared using the *t*-test or Mann-Whitney *U* test, as appropriate, based on the distribution of the data. For categorical variables, the frequencies and percentage within each category will be calculated. Groups will be compared using the chi-square or Fisher’s Exact test, as appropriate, based on the expected counts. All available data will be summarized. Demographics, preoperative, perioperative, and postoperative parameters will be reported and compared for both groups. Descriptive statistics will be presented to describe the trial results. Missing data will be evaluated by the investigators, and appropriate action will be undertaken*.* In case of skewed baseline data between groups, results will be corrected for this data.

Our primary analysis will focus on the effectiveness and safety of the C-Qur film between the treatment group and the control group. A chi-square or Fisher’s Exact test will be performed to assess a significant improvement in the incidence, on the Zühlke score of adhesions underneath the incision site, underneath the loop ileostomy or loop/split colostomy site, and for the extent of adhesions underneath the loop ileostomy or loop/split colostomy site as well. The extent of adhesions underneath the incision site and the time needed for takedown of the ileostomy or loop/split colostomy will be compared using a *t*-test. To assess the safety of the C-Qur film the total incidence of postoperative complications will be compared using a *t*-test or a Mann-Whitney *U* test. The mortality and the incidence for each separate complication will be compared using a chi-square or Fisher’s Exact test.

Subgroup analysis will be performed on laparoscopic versus open colorectal resection, and on ileostomy versus colostomy for the incidence, extent and severity of adhesions as described above. Since data collection of the outcome parameters is not blinded when the same surgeon who performed the index procedure also takes down the ostomy, subgroup analysis will be performed for patients with and without blinded data collection of outcome parameters.

### Reporting

The CLIPEUS trial findings will be reported in concordance with the Consolidated Standards of Reporting Trials (CONSORT) checklist [23].

## Discussion

Results of this study add to the evidence on the use of anti-adhesive barriers in open and laparoscopic ‘hand-assisted’ colorectal surgery. Although the ultimate objective of adhesion prevention is to reduce the clinical consequences of adhesions, we chose incidence of adhesions to the incision site as primary outcome measure. Clinical outcomes such as small bowel obstruction, secondary infertility and adhesiolysis related complications are considered multifactorial and difficult to interpret [24]. Incidence of adhesions at repeat surgery is believed to be the most valuable surrogate endpoint for clinically relevant adhesion prevention, since small bowel obstruction and adhesiolysis at repeat surgery are not likely to occur when complete adhesion reduction in a patient is accomplished. No adhesion under the incision site in particular will benefit patients at re-laparotomy or re-laparoscopy. Recent evidence from our group emphasizes the large disease and socioeconomic burden of adhesions needing lysis at subsequent abdominal surgery [5].

To be able to evaluate incidence of postoperative adhesions, a second-look surgery model is required, also because noninvasive methods such as cine-magnetic resonance imaging (MRI) have not been validated measuring adhesion reduction [25]. Colorectal resection with temporary loop ostomy is an obvious choice, since colorectal surgery is frequently performed and known for its relatively high incidence of adhesion-related complications [26]. We will include patients undergoing resection for benign or malignant indication. Malignancy is the largest indication for colorectal resection, and since (disease-free) survival has strongly increased, life time risk of adhesion-related complications has increased correspondingly.

In this study, both laparoscopic and open colorectal resections will be included. In many countries, laparoscopic colorectal resection for benign and malignant diseases has gained popularity [27]. Thus, performing only an open colonic resection study would lower the generalizibility of the results on adhesion prevention. Laparoscopic technique is accompanied by less tissue trauma. Hence, it is suggested that laparoscopic colorectal surgery results in fewer adhesions. A recently published population-based register study specifically addressed readmission rate for clinically apparent adhesions after colorectal surgery, comparing the open and laparoscopic approach [28]. Of the total of 187,148 patients included, 11,013 (5.9%) had laparoscopic resection. With a median follow-up of 31.8 months, overall adhesion-related readmission rate was 8.1%; 8.2% after an open approach versus 6.3% after a laparoscopic approach (*P* <0.001). An important limitation of this study was the higher percentage of emergency operations in the open group compared to the laparoscopic group. The most common underlying disorder for an emergency laparotomy (that is, peritonitis) has a higher adhesion formation propensity. Despite the relative reduction of 23% in the re-admission rate, it should be concluded that clinically relevant consequences of adhesions are substantial also after laparoscopic surgery. A subgroup analysis for open versus laparoscopic resection will be conducted to control for the difference in surgical technique and concomitant adhesion formation.

For the sake of safe and secure placement of the adhesion barrier, only patients undergoing laparoscopic resection with a specimen extraction incision of at least 6 cm will be included in our study. This minimal incisional length was chosen based on previous experience with the C-Qur™ Film. The availability of different sizes of the film and the tissue adherence only at one site improve the placement in narrow spaces. These characteristics provide potentially better handling when compared to the commonly used HA/CMC barrier film in open colorectal surgery.

## Trial status

This trial has been approved by the official Independent Review Board Nijmegen (2013/470) [15]. Inclusion has not started.

## Abbreviations

AE: adverse events
ASA: American Society of Anesthesiologists
CONSORT: Consolidated Standards of Reporting Trials
CRF: case report form
CRP: C-reactive protein
DASI: duke activity status index
DSMB: Data Safety Monitoring Board
HA/CMC: hyaluronate/carboxymethylcellulose
INR: international normalized ratio
K: potassium
MRI: magnetic resonance imaging
Na: sodium
POI: postoperative ileus
PT: prothrombin time
RCRI: Revised Cardiac Risk Index
SF-36: short-form 36
SSI: surgical site infection
VAS: Visual Analog Scale.
